# How to Boil Water

**Published:** 1885-03

**Authors:** 


### HOW TO BOIL WATER.

I must tell you the old story of how the late Charles Delmonico used to talk about the new hot water cure. He said, the Delmonicos were the first to recommend it to the guests, who complained of having no appetite. "Take a cup of hot water and lemon and you will feel better," was the formula adopted, and the cup of hot water and lemon was simply a little hot water with a drop of lemon juice in it to take away the insipidity. For this antibilious remedy the caterers charged the price for their best liquors—twenty-five cents or more—and it was certainly a wiser way to spend small change than in alcohol. "Few people know how to cook water." Charles used to affirm. "The secret is in putting good, fresh water into a neat kettle, already quite warm, and setting the water to boiling quickly, and then taking it right off for use in tea, coffee, or other drinks, before it is spoiled. To let it steam and simmer and evaporate until the good water is in the atmosphere, and the lime and iron and dregs only left in the kettle—bah! that is what makes a great many people sick, and it is worse than no water at all." Every lady who reads the recipe of a great and careful cook should never forget how to cook water.

